# A simple hydrofluoric acid-free pressurized-cavity microwave-assisted acid digestion method for determination of impurity elements in recycled carbon fibers with ICP-MS

**DOI:** 10.1007/s44211-026-00886-1

**Published:** 2026-03-05

**Authors:** Tetsuya Nakazato, Yoshiki Makino

**Affiliations:** Environmental Management Research Institute, National Institute of Advanced Industrial Science and Technology (AIST), 16-1 Onogawa, Tsukuba, Ibaraki 305-8569 Japan

**Keywords:** Carbon fiber, Recycled carbon fiber, Elemental analysis, Metal, ICP-MS

## Abstract

A simple method was proposed for the determination of 13 impurity elements (Na, Mg, Al, K, Ca, Ti, Cr, Fe, Co, Ni, Cu, Zn, and Pb) in recycled carbon fibers (rCFs) using pressurized-cavity microwave-assisted acid digestion without hydrofluoric acid (HF) combined with inductively coupled plasma mass spectrometry. Elevating the digestion temperature to 280 °C under pressurized conditions facilitated the dissolution of the target elements in rCF, even when present in refractory forms. Because certified reference materials of rCF samples for determining impurity elements were unavailable, the validity of the proposed method was confirmed by comparing its analytical results with those obtained using a HF-added digestion method, which completely decomposes silica, followed by the dissolution of the bound target elements. The proposed method was further validated by analyzing carbon- and metal-based certified reference materials and metal oxides as analogous to the refractory components of rCF. However, the limited decomposition of refractory rutile-type titania suggests an upper limit of 890 mg/kg for titanium determination using the proposed HF-free method. The limits of detection ranged from 0.0014 mg/kg for cobalt to 0.72 mg/kg for potassium. Ten of the 13 elements showed lower limits than those of the HF-added method because of less contamination. Operationally, the HF-free method is safer and simpler because it avoids the use of hazardous HF and does not require HF-elimination treatment.

**Graphical abstract:**

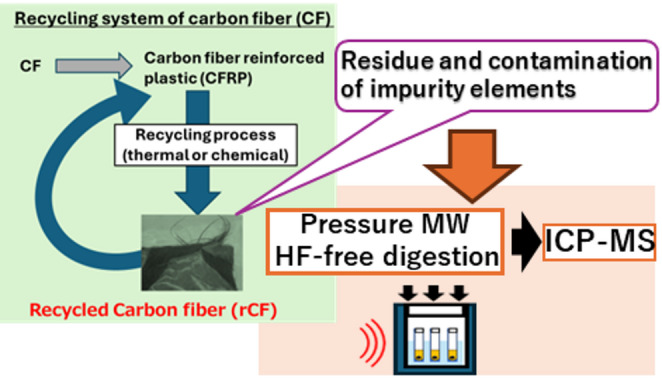

**Supplementary Information:**

The online version contains supplementary material available at 10.1007/s44211-026-00886-1.

## Introduction

Recycled carbon fiber (rCF) is extracted from discarded carbon fiber reinforced composites (CFRCs), such as carbon fiber reinforced plastic (CFRP) and multi-material structured CFRP containing carbon fiber (CF), polymers, and metals [1–3]. The use of rCF has advantages in terms of cost and reduction of CO_2_ emissions throughout its life cycle. Therefore, to manage the safety and quality of the produced rCFs, it is necessary to establish analytical methods for the impurity elements contained in rCF. rCF is a carbon-based mixture containing impurity elements that are derived from the main components, that is, CF, residuals of the plastics, and the multi-material structure, as well as from chemical agents and catalysts used in the extraction of CF during recycling operations. Analysis of 13 impurity elements (Si, Al, Ca, Ti, V, Cr, Fe, Ni, Cu, Zn, Y, Mo, and Sb) in a CF sample was performed using inductively coupled plasma mass spectrometry (ICP-MS) with the dissolution of hydrochronic acid after ashing and conventional closed-vessel microwave-assisted acid digestion using nitric acid and hydrogen peroxide [4]. However, this study did not evaluate the accuracy, reproducibility, or applicability of rCF samples. There are also studies on the analysis of refractory carbon-based materials that are similar to the components of CF. CF consists of crystalline and non-crystalline carbon; therefore, graphite and coal can be considered as model materials for these respective carbon components. A closed-vessel microwave-assisted acid digestion of the sample using nitric acid and hydrogen peroxide successfully determined the ten elements (Ba, Ca, Cr, Cu, Fe, Mg, Mn, Na, Ni, and Zn) by ICP optical emission spectrometry (ICP-OES) and ICP-MS even under incomplete decomposition of graphite [5]. Additionally, six elements (Co, Fe, Ni, W, Y, and Zr) were successfully determined by a pressurized-cavity microwave-assisted acid digestion method that elevated the digestion temperature for decomposition of the sample. However, this required complicated operations and hazardous and interfering acid reagents, such as hydrofluoric acid (HF) and sulfuric acid [6]. In the analysis of a certified reference material (CRM) of coal by ICP-OES and ICP-MS, 17 elements (Li, Be, Na, Al, K, Fe, V, Cr, Mn, Co, Ni, Cu, Zn, As, Se, Cs, and Pb) were successfully determined by closed-vessel microwave-assisted acid digestion using nitric acid and hydrogen peroxide [7], in which the absence of HF led to incomplete decomposition of the silica components. An integrated method of oxidative pyrolysis and microwave-assisted acid digestion using NH_4_F as a substitute for HF combined with ICP-OES and ICP-MS measurements successfully identified 34 elements (Be, Na, K, Ca, Al, Mg, Sc, Ti, V, Fe, Cr, Mn, Co, Ni, Cu, Zn, As, Rb, Sr, Mo, Cd, Sb, Cs, Ba, W, Tl, Pb, Th, U, and rare-earth elements) in CRMs of coal [8]. In the case of other non-crystalline carbon materials, such as charcoal and carbon black, direct solid sampling high-resolution continuum source graphite furnace atomic absorption spectrometry was used to determine seven heavy metals (Cr, Cu, Fe, Mn, Mo, Ni, and V) [9].

As for the metal elements that were originally derived from the multi-material CFRC, steel is a representative material with characteristics that make it difficult to decompose and determine. The impurity elements (e.g., B, Mg, Al, S, Si, P, Ca, Ti, V, Cr, Mn, Co, Cu, Ni, Mo, and W) in the steels were determined using ICP-OES or ICP-MS with closed-vessel microwave-assisted acid digestion using HF with the addition of phosphoric acid, sulfuric acid, or perchloric acid for complete decomposition, or with pressurized-cavity microwave-assisted acid digestion using HF [10–13].

This study proposes a simple method for determining impurity elements in rCF. This method consists of HF-free pressurized-cavity microwave-assisted acid digestion and ICP-MS measurement. Thirteen elements (Na, Mg, Al, K, Ca, Ti, Cr, Fe, Co, Ni, Cu, Zn, and Pb) were targeted to assess the harmfulness and quality of rCF and to be found in real rCF samples. The pressurized-cavity digestion increased the temperature to improve the dissolution of the target elements from rCF without the use of HF, which increases the hazardous risk, damages the glass interface that is generally used for ICP-MS instruments, and then requires additional complicated operations for elimination (e.g., evaporation or masking of HF) or an expensive interface of fluoropolymer and platinum for direct analysis without elimination. Because reference materials of rCF for determining impurity elements were unavailable, the proposed method was validated by comparison with an HF-added closed-vessel microwave-assisted acid digestion method and by analyzing carbon- and metal-based CRMs as analogs of the refractory components of rCF.

## Experimental

### Regents and materials

Nitric acid (70% (w/w)), hydrogen peroxide (30% (w/w)), and hydrofluoric acid (48% (w/w)) were of ultrapure grade (Kanto Chemical, Japan). Ultra-pure water was prepared using a Milli-Q™ Integral system with the Element unit (Millipore, USA) was used throughout for preparing solution and rinsing digestion vessels and bottles. A standard stock solution (XSTC-331, SPEX, USA, 10 mg L^− 1^) and a titanium stock solution (for ICP analysis, FujiFilm-Wako, Japan, 1000 mg L^− 1^) were used to prepare calibration solutions for ICP-MS by dilution with diluted nitric acid. Standard stock solutions of the multi-element and single element (sodium, magnesium, aluminum, potassium, calcium, titanium, iron, nickel, and zinc, for ICP analysis, 1000 mg L^− 1^) were used as the spiking solutions for the recovery tests. The CRMs (NIST 1632d, trace elements in coal, USA; BAM-S009, medium-purity graphite powder, Germany, JSS 651 − 16, SUS304 stainless steel, Japan; and JSS 171-8, carbon steel, Japan) were used to validate the proposed method. Metal oxides (α-aluminum oxide, rutile- and anatase-type titanium oxides, chromium oxide, iron(III) oxide (hematite), copper(II) oxide, nickel(II) oxide, and zinc oxide] (> 99%, fine powder; Koujyundo Kagaku, Japan) were also used. A filter unit (Millex-HV, 0.45 μm pore size, polyvinylidene difluoride membrane, Merck, Germany) and a syringe of polypropylene were used for the separation of residue when it was observed after digestion of the sample. The filters and syringes were cleaned using dilute nitric acid and rinsed with water.

### Recycled carbon fiber and carbon fiber samples

Two types of recycled carbon fiber samples (rCF1 and rCF2) were used. The rCF samples were recovered from aircraft scraps through thermal treatment. The rCF1 was commercially available (ELG Carbon Fiber Ltd., Carbiso C, IMS56P-03/10) and was used. The rCF2 and new carbon fiber (CF1) were supplied by the manufacturers of recycled carbon fiber and carbon fiber, respectively. The lengths of the rCF1 and CF1 samples were both < 10 mm. The lengths of the rCF2 samples were adjusted to less than 10 mm by cutting them using ceramic scissors. To improve analytical precision, rCF and CF samples (a few grams) were pulverized using a rod mill (MB3000, Yasui Kikai, Japan) with a zirconia rod and a polycarbonate container at 3000 rpm for 60 s. The pulverized samples were collected in clean polypropylene bottles.

### Instruments

#### Microwave digestion systems

Microwave-assisted acid digestion of the samples was performed using a pressurized-cavity microwave digestion system (Multiwave 7000, Anton Paar, Austria) or a conventional closed-vessel microwave digestion system (Multiwave Pro, Anton Paar). The conceptual diagrams of the two systems are shown in Fig. S1. The former system was used for HF-free pressurized-cavity microwave-assisted acid digestion and was equipped with quartz digestion vessels with tetrafluoromethane-modified (TFM) caps in a tube rack within a liner of polytetrafluoroethylene (PTFE)-TFM in a pressurized-cavity of alloy. The latter system was equipped with a digestion rotor and utilized either TFM digestion vessels covered with ceramic vessel jackets for HF-added closed-vessel microwave-assisted acid digestion or quartz digestion vessels for HF-free closed-vessel microwave-assisted acid digestion.

#### ICP-MS instrument

The dissolved elements in the digested solution were analyzed using an ICP-MS spectrometer (Agilent 8900, Agilent, Japan). A concentric nebulizer and a double-pass quartz spray chamber were used. The operating conditions are listed in Table S1.

### Procedure

#### Microwave-assisted digestion

After optimizing the conditions, the procedure for the proposed HF-free pressurized-cavity microwave-assisted acid digestion method for rCF samples is described below. A 0.0125 g sample of rCF was placed in a digestion vessel, and 5 mL of nitric acid and 1 mL of hydrogen peroxide were added. The vessel was then placed in a rack within a liner in a cavity. The liner was filled with 155 mL of 0.5 M nitric acid as the load solution for uniform heating and pressure stabilization. The cavity was pressurized by introducing nitrogen gas (purity > 99.9%) to 3 MPa). The heating program was controlled by monitoring the liner temperature. It was carried out in two stages: a ramp stage to raise the temperature from room temperature to 280 °C for 40 min, followed by a hold stage for 50 min. After digestion, the vessels were cooled below 80 °C and then the clear sample digest was transferred to a polypropylene bottle and diluted with water to adjust the nitric acid concentration for subsequent ICP-MS analysis.

The procedure for the HF-added closed-vessel microwave-assisted acid digestion method is described below. A 0.05 g sample of rCF was placed in a digestion vessel, and then 5 mL of nitric acid, 1 mL of hydrogen peroxide, and 0.2 mL were added. A predetermined power program was used to control the heating [7]. Typically, the temperature was raised to 200 °C within the first 30 min and to a peak temperature of 200–220 °C within the next 40 min, with a maximum pressure of 5.8 MPa. After cooling to below 50 °C, evaporate the clear digest sample to a small drop of thick liquid residue in a perfluoroalkoxyalkane (PFA) jar on a hot plate at 90 °C in a HEPA‑filtered clean bench. The residue was dissolved in dilute nitric acid for ICP-MS analysis. To investigate HF-free closed-vessel microwave-assisted digestion under lower temperature conditions (210–230 °C), quartz vessels were used. The heating program was controlled in the same manner as that for the HF-added method. The difference was that the maximum pressure was set to 7.4 MPa, which increased the peak temperature to 210–230 °C. The ramp and hold times were the same as those in the HF-supplemented method: 30 min and 40 min, respectively. When residue was observed after the HF-free digestions in the case of analysis of the rCF at below 250 °C or analysis of the graphite CRM, the digested solution was filtrated using a filter unit. Alternatively, the residue from the digested solution of metal oxides (e.g., titania) was separated by centrifugation at 2,270 ⋅*g* for 10 min) and the supernatant was collected. The filtrate or supernatant was diluted with water and used for the ICP-MS analysis. Throughout the experiment, the polypropylene sample bottles and PFA jars were cleaned by immersing them in a 10% (w/w) nitric acid solution overnight, then in ultrapure water overnight, and finally drying. The digestion vessels were cleaned using a mixture of 60% (w/w) nitric acid and 5% (w/w) hydrochloric acids under pressurized-cavity microwave digestion at 220 °C for 10 min or closed-vessel microwave digestion at approximately 200 °C for 40 min. Finally, the vessel was washed with water and dried.

#### ICP-MS analysis

ICP-MS analysis of the dissolved elements in the digested solution was performed under an optimized condition using a tuning solution containing Li, Co, Y, Ce, and Tl. The 13 elements (Na, Mg, Al, K, Ca, Ti, Cr, Fe, Co, Ni, Cu, Zn, and Pb) were determined using an internal calibration method and collision/reaction cell gas modes with helium, hydrogen, or oxygen for single mass spectrometry (MS) or MS/MS detection. The selected isotopes and MS modes are listed in Table S1.

## Results and discussion

### Pressurized-cavity and closed-vessel microwave-assisted hydrofluoric acid-free digestion of rCF

We investigated HF-free microwave-assisted acid digestion conditions to determine the impurity elements in the rCF samples. The target elements selected for this study were 13 elements: Na, Mg, Al, K, Ca, Ti, Cr, Fe, Co, Ni, Cu, Zn, and Pb. These elements were detected in the rCF samples and considered for the evaluation of harmfulness and material quality. The rCF1 sample was used for the initial examination because of its high elemental concentrations, based on preliminary tests. The rCF1samples were less than 10 mm in length and were directly digested without pulverization to minimize elemental contamination. First, the HF-free closed-vessel microwave-assisted digestion of the rCF1 sample was tested according to the conditions that successfully dissolved and determined trace elements, mainly heavy metals, in coal [7]. The amounts of rCF1, nitric acid, and hydrogen peroxide were 0.05 g, 2.5 ml and 0.5 ml, respectively. The ratio of the rCF sample to reagent mixture was 0.017 (w/v). The resultant digestion temperatures were raised to 210–230 °C. The target 13 elements were detected (Fig. S2); however, black-colored residues were observed after digestion. To further decompose the sample, the sample-to-reagent ratio was reduced by half (0.008 w/v, 0.025 g/3 ml). Although the dissolution of Ti increased (Fig. S2), residue was still observed. We attempted to improve the decomposition and dissolution of the target elements by elevating the temperature using a pressurized-cavity microwave digestion system because the closed-vessel system disabled the elevation owing to the limitation of the maximum operation pressure. Increasing the temperature to 250 °C and slightly increasing respectively the digestion ramp and holding time to 40 and 50 min further dissolved alkaline earth metals (Mg and Ca) at the same sample-to-reagent ratio of 0.008 (w/v), i.e. 0.05 g/6 ml (Fig. S2). Further reduction of the sample-to-reagent ratio to 0.002 (w/v), that is, 0.0125 g/6 ml, resulted in an increase in Ti concentration only (Fig. S2), while the residue was still observed. Further increasing the temperature to 280 °C, no residues were observed. The concentrations of nine elements (Na, Mg, Al, Ca, Ti, Cr, Fe, Co, and Ni) significantly increased, whereas there was no increase or a slight increase in the concentrations of K, Cu, Zn, and Pb (Fig. S2). It is also noted that direct comparison of temperature values is difficult between the pressurized-cavity and closed-vessel microwave digestion systems, because the former case measured the temperature of the liner filling with the load solution outside the digestion vessel while the latter case measured the temperature of digestion vessel filling with the sample solution using an infrared absorption sensor. However, the increase in the digestion temperature is probably certain in the case of pressurized-cavity-type system, because both sample decomposition and element dissolution were promoted. The repeatability results showed that the relative standard deviation (RSD) for eight elements (Mg, Al, Cr, Fe, Co, Ni, Zn, and Pb) was greater than 30%. This indicates that the rCF samples were heterogeneous. The pulverization of the rCF sample using a rod mill improved the RSD values of all 12 target elements, except for K. The resultant RSD values of 12 elements, except Ni, ranged from 1 to 10% (Table 1). The Ni RSD remaining at 20% implies inherent limitations of this grinding technique regarding the elemental homogenization. The average values after pulverization were also generally comparable to the values before pulverization, suggesting that this treatment did not significantly affect the dissolution and contamination of the elements.

**Table 1:** Concentrations of impurity elements in rCF and CF samples by HF-free pressurized-cavity microwave-assisted acid digestion at 280 °C and HF-added closed-vessel microwave-assisted acid digestion at 200–220 °C

Element	HF-free pressurized-cavity digestion		HF-added closed-vessel digestion		Ratio of average concentrations by HF-free and HF-added digestion (%)	
	Average ± SD (mg/kg)	RSD (%)	Average ± SD (mg/kg)	RSD (%)		
*rCF1*						
Na	58.3 ± 0.5	1	58.2 ± 0.3	0.5	100	
Mg	120 ± 2	2	133 ± 10	10	90	
Al	787 ± 60	8	839 ± 90	11	94	
K	36.7 ± 4.0	10	34.4 ± 2.0	7	107	
Ca	599 ± 9	1	569 ± 30	5	105	
Ti	429 ± 4	1	413 ± 8	2	104	
Cr	17.5 ± 1.8	10	17.7 ± 2.0	10	99	
Fe	177 ± 10	8	183 ± 20	10	97	
Co	0.877 ± 0.020	2	0.976 ± 0.09	9	90	
Ni	4.57 ± 0.91	20	4.41 ± 0.80	18	104	
Cu	4.07 ± 0.40	10	4.02 ± 0.30	8	101	
Zn	3.08 ± 0.25	8	2.97 ± 0.10	5	104	
Pb	0.216 ± 0.01	5	0.200 ± 0.006	3	108	
*rCF2*						
Na	5.39 ± 0.41	8	4.93 ± 0.15	3	109	
Mg	6.42 ± 0.57	9	6.58 ± 0.26	4	98	
Al	21.8 ± 1.2	5	20.7 ± 0.5	2	105	
K	2.54 ± 0.96	38	2.69 ± 0.13	5	94	
Ca	97.9 ± 2.1	2	99.2 ± 1.8	2	99	
Ti	25.0 ± 1.0	6	26.2 ± 1.2	5	95	
Cr	0.358 ± 0.04	11	0.363 ± 0.044	12	99	
Fe	23.4 ± 1.7	7	25.9 ± 2.4	9	90	
Co	1.32 ± 0.04	3	1.31 ± 0.033	3	101	
Ni	3.32 ± 0.08	2	3.34 ± 0.096	3	99	
Cu	12.2 ± 0.2	2	12.1 ± 0.18	2	101	
Zn	1.35 ± 0.46	34	< LOD (0.92)		n.d.	
Pb	0.149 ± 0.005	3	0.143 ± 0.003	2	104	
*CF1*						
Na	8.17 ± 0.32	4	8.42 ± 0.38	5	97	
Mg	0.503 ± 0.11	21	< LOD(0.6)		n.d.	
Al	0.311 ± 0.18	57	< LOD(1.3)		n.d.	
K	7.25 ± 0.21	3	6.33 ± 0.28	4	114	
Ca	7.77 ± 0.13	2	8.24 ± 0.88	11	94	
Ti	< LOD(0.05)		< LOD(0.04)		n.d.	
Cr	0.168 ± 0.021	12	0.179 ± 0.027	15	94	
Fe	0.799 ± 0.29	36	< LOD(7.1)		n.d.	
Co	0.00953 ± 0.0021	22	< LOD(0.02)		n.d.	
Ni	< LOD(0.05)		< LOD(0.03)		n.d.	
Cu	0.0206 ± 0.012	57	< LOD(0.06)		n.d.	
Zn	< LOD(0.49)		< LOD(0.92)		n.d.	
Pb	0.0107 ± 0.00058	5	< LOD(0.01)		n.d.	

n.d.: not determined. < LOD: Below detection limit. Values were obtained from three repetitive analyses

Next, we verified whether a decrease in sample-to-reagent ratio (0.002 (w/v)) and the pulverization of rCF samples promoted the dissolution of elements under closed-vessel microwave digestion conditions at lower temperatures (210–230 °C) and a slightly shorter digestion ramp and hold time (30 and 40 min). The reduction of sample-to-reagent ratio in conjunction with pulverization has facilitated the sufficient dissolution of eight elements (K, Ti, Cr, Fe, Ni, Cu, Zn, and Pb) for determination even under the condition of the lower temperatures and shorter digestion times (Fig. S2). However, these treatments were found to be inadequate in sufficiently dissolving five elements (Na, Mg, Al, Ca, and Co). Furthermore, residue was observed. Consequently, the higher temperature at 280 °C and longer digestion time are necessary for sufficient dissolution and that the chemical forms of the elements appear to be refractory.

### Comparison of hydrofluoric acid-free pressurized-cavity and hydrofluoric acid-added closed-vessel microwave-assisted acid digestion

The validity of the proposed method of HF-free pressurized-cavity microwave-assisted acid digestion at 280 °C was evaluated by comparing its analytical results with those obtained using a HF-added closed-vessel microwave-assisted acid digestion method. This was due to the fact that CRMs of rCF were unavailable and the HF-added method can achieve complete decomposition of silica, which is only partially decomposed by HF-free digestion [7, 12, 14, 15], followed by dissolution of the target elements that are bound to the oxide. For instance, the concentration of potassium in a coal CRM obtained by HF‑added digestion using a closed‑vessel microwave digestion system even with lower temperature conditions (220–240 °C) agreed with the certified value, whereas the concentration obtained by HF‑free digestion using the same system and temperature conditions was lower [7]. The pulverized rCF1 sample was decomposed even under the HF-added condition at a lower temperature of 200–220 °C with a higher sample-to-reagents ratio of 0.008 (w/v), i.e. 0.05 g/6.2 mL (5 mL HNO₃ + 1 mL H₂O₂ + 0.2 mL HF). No residue was observed after post-treatment of evaporation or dilution with diluted nitric acid. The concentration values of the 13 target elements obtained by the proposed HF-free digestion with sample-to-reagent ratio of 0.002 (w/v) at 280 °C showed good agreement with the HF-added digestion method (90–108%), as shown in Table 1. This result demonstrated that the proposed HF-free method achieved sufficient dissolution of the target elements, including those bound to silica. In addition, to verify the applicability of the rCF samples, we analyzed another rCF sample (rCF2) obtained from a different raw CFRP and recycling process. No residue was observed in either method, with or without adding of HF. The results for 11 elements (Na, Mg, Al, Ca, Ti, Cr, Fe, Co, Ni, Cu, and Pb) obtained by the proposed HF-free method showed good agreement with the HF-added method (90–109%), as shown in Table 1. Furthermore, the RSDs for the proposed HF-free method in repetitive analyses were satisfactory, ranging from 2% to 12%. Potassium also showed average values were equivalent to those obtained using the HF-added method, but the RSD exceeded 30% owing to concentrations near the limit of quantification (LOQ), as described below. Zinc was detectable by only the proposed HF-free method, as it is less contaminated due to the absence of post-treatment (such as evaporation) required in the HF-added method. However, given that the concentration was near the LOQ, a higher RSD value was observed.

Furthermore, we verified its applicability to CF, which is the main component of rCF, and its high purity was suitable for determination at low concentrations. No residue was observed, and the four elements (Na, K, Ca, and Cr) showed good agreement with the HF-added method (94–114%), as shown in Table 1. Six elements (Mg, Al, Fe, Co, Cu, and Pb) were determined using only the proposed HF-free method with lower LOQs; however, the repeatability was low because their concentrations were near the LOQ.

### Analytical performance

The limits of detection (LODs) for 13 elements in the proposed HF-free pressurized-cavity microwave-assisted acid digestion method with ICP-MS at a sample-to-reagent ratio of 0.002 (w/v) were examined. The LOD was defined as three times the standard deviation of repetitive analyses (*n* = 5) in blank tests without samples. The LOD was ranged from 0.0014 mg/kg for Co to 0.72 mg/kg for K. (Table 2) The LODs of 10 elements (Na, Mg, Al, Ca, Ti, Fe, Co, Cu, Zn, and Pb) were 0.01 to 0.7 times lower than those of HF-added closed-vessel microwave-assisted acid digestion method. The LODs of the other three elements (K, Ti, and Ni) were only slightly higher (1.9, 1.4, and 1.6 times) than those obtained using the HF-added method. The proposed HF-free method yielded excellent LOD results even under unfavorable dilution conditions. For the ICP-MS analysis, the samples were diluted 16-fold compared to the HF-added method. This was due to a relatively lower sample-to-reagent ratio (0.25) during digestion and 4-fold higher dilution rate for the digested rCF sample solution before the ICP-MS measurement because of the reduced nitric acid concentration resulting from the addition of water. A closed and clean digestion operation without post-treatment (e.g., evaporation) is considered to reduce elemental contamination.

**Table 2:** Limits of detection of HF-free pressurized-cavity and HF-added closed-vessel microwave-assisted acid digestion methods with ICP-MS

Element	Limit of detection (mg/kg)	
	HF-free pressurized-cavity digestion method	HF-added closed-vessel digestion method
Na	0.12	0.92
Mg	0.41	0.57
Al	0.10	1.3
K	0.72	0.39
Ca	0.47	1.5
Ti	0.055	0.040
Cr	0.046	0.056
Fe	0.10	7.1
Co	0.0014	0.023
Ni	0.051	0.031
Cu	0.010	0.060
Zn	0.49	0.92
Pb	0.0019	0.013

Recovery tests were performed to further evaluate elemental loss and contamination during the proposed digestion operation. Assuming analysis in the low-concentration range, where these influences are pronounced, the CF1 sample with the lowest elemental concentration was used in this study. The recovery rates were evaluated by adding elemental standard solutions to the CF1 samples in a digestion vessel. Since there are many kinds of elements with wide ranges of concentration and analytical precision, the amount to be added to each element was defined as follows: (1) For the 3 elements Na, K, and Ca above 1ppm, high precision is expected with RSD < 10%, so they were added in amounts 1–2 times the sample concentration; (2) For the five elements (Mg, Al, Cr, Fe, and Cu) below 1ppm with RSD > 10% and the undetectable three elements (Ti, Ni, Zn), they were added in amounts 3–17 times LOQ; (3) For Co and Pb below 0.1ppm, they were added as 0.2 ppm that is the same concentration added to Cu because a multi-element solution containing Cu was added at 2). The concentrations of each element ranged from 0.2 to 20 ppm. The average recovery was 91–110% (Table 3), indicating that even in this low concentration range, there was little influence of elemental loss and contamination.

**Table 3:** Results of recovery tests using the spiked CF1 samples

Element	Spiked concentration (mg/kg)	Recovery (%)	Information of spiking amounts
Na	10	101 ± 2	1-fold the sample concentration (SC)
Mg	20	103 ± 2	17-fold LOQ
Al	4.0	104 ± 1	11-fold LOQ
K	10	99 ± 9	1-fold SC
Ca	20	92 ± 7	2-fold SC
Ti	0.5	96 ± 7	3-fold LOQ
Cr	1.0	96 ± 3	7-fold LOQ
Fe	1.5	110 ± 7	4-fold LOQ
Co	0.2^*a*^	96 ± 6	39-fold LOQ
Ni	1.0	110 ± 3	4-fold LOQ
Cu	0.2	91 ± 8	5-fold LOQ
Zn	10	95 ± 6	6-fold LOQ
Pb	0.2^*a*^	110 ± 5	28-fold LOQ

*a*: Spike concentration was the same as that of copper obtained by spiking a multi-element solution. LOQ: limit of quantification. Recovery values were obtained from three repetitive analyses

### Validation using certified reference materials as analogues of refractory components of rCF

To further verify the proposed HF-free pressurized-cavity microwave-assisted acid digestion method for the target elements, we analyzed CRMs that were similar to the refractory components of rCF and evaluated the results for each element by comparing them with certified values. First, we investigated the applicability of amorphous carbon, which is one of the main components of CF in rCF, to a CRM of coal (NIST SRM 1632d), as shown in Table 4. Coal is also known as one of the raw materials for pitch-based CF. The concentration values for all the 13 target elements (Na, Mg, Al, K, Ca, Ti, Cr, Fe, Co, Ni, Cu, Zn, and Pb) and additional 12 elements (S, V, Mn, As, Se, Rb, Sr, Cd, Cs, Ba, Th, and U) were in agreement with the certified, reference, and information values (85–106%). In particular, the higher temperature condition at 280 °C significantly improved the dissolution and the determination of Ti (average value was 85% (*n* = 3)), comparing with the results obtained under a closed-vessel and lower-temperature digestion condition at 210–230 °C (average value was 45% (*n* = 3)). Next, a graphite CRM (BAM-S009) was analyzed as an analog of the crystalline carbon of CF in the rCF (Table 4). The residue was observed after the HF-free digestion. However, seven target elements (Ca, Ti, Cr, Fe, Co, Ni, and Cu) and additional 3 elements (S, V, and As) were sufficiently dissolved and were successfully determined. Six target elements (Na, Mg, Al, K, Zn, and Pb) and two other 2 elements (Mn and Cd) were below the LODs. It is noted that the LOD of Pb was increased to 0.06 mg/kg by the contamination from filtration of the residue before the ICP-MS measurements. Such incomplete decomposition of graphite samples but sufficient dissolution of the target elements for determination has also been reported in a HF-free closed-vessel microwave-assisted acid digestion method under a lower temperature condition [5]. The eight target elements (Ca, Cr, Fe, Ni, Cu, Na, Mg, and Zn) with concentrations higher than those used in this study were successfully determined. Based on the results, the proposed HF-free microwave-assisted digestion condition at 280 °C could determine at least 10 of the 13 target elements (Na, Mg, Ca, Ti, Cr, Fe, Co, Cu, Ni, and Zn) in the graphite. Other elements (K, Pb, and Al) could not be validated because the concentrations were below the LOQs. The proposed HF-free method for target elements in graphite is simpler and does not require the use of multiple hazardous and interfering reagents (HF, sulfuric acid, and perchloric acid) for the complete decomposition of the sample [6].

**Table 4:** Analyses of certified reference materials of coal and graphite (NIST 1632d and BAM-S009) as carbonous analogues of rCF components

Element	Certified or reference or information value ± uncertainty (mg/kg)	Measured average value ± SD (mg/kg)	RSD (%)	Measured average value /certified or reference or information value (%)
*NIST 1632d*				
Na	296.9 ± 4.2	295 ± 18	6	99
Mg (r)	390 ± 6	359 ± 6	2	92
Al (r)	9,120 ± 50	8,780 ± 900	10	96
S	14,620 ± 740	13,400 ± 200	1	91
K	1094 ± 26	982 ± 1	0.1	90
Ca^®^	1440 ± 30	1420 ± 150	11	99
Ti	477 ± 10	406 ± 31	8	85
V	23.7 ± 0.1	23.5 ± 2.3	10	99
Cr (r)	13.7 ± 0.1	14.5 ± 1.3	9	106
Mn (r)	13.1 ± 0.4	13.5 ± 1.6	12	103
Fe	7,490 ± 160	7,680 ± 170	2	103
Co	3.424 ± 0.048	3.38 ± 0.25	7	99
Ni (i)	10	10.6 ± 1.0	9	106
Cu	5.83 ± 0.31	6.12 ± 0.5	8	105
Zn (r)	12.9 ± 1.2	11.8 ± 0.4	3	92
As (r)	6.1 ± 0.2	5.95 ± 0.07	1	97
Se (r)	1.29 ± 0.03	1.20 ± 0.06	5	93
Rb	7.36 ± 0.2	7.48 ± 0.75	10	102
Sr	63.5 ± 1.2	63.1 ± 2.9	5	99
Cd (r)	0.08 ± 0.01	0.0724 ± 0.0100	14	90
Cs (r)	0.598 ± 0.006	0.601 ± 0.059	10	100
Ba	40.42 ± 0.89	39.6 ± 2.9	7	98
Pb	3.845 ± 0.042	3.99 ± 0.23	6	104
Th	1.428 ± 0.035	1.38 ± 0.12	9	97
U	0.517 ± 0.012	0.495 ± 0.062	13	96
*BAM-S009*				
Na	0.32 ± 0.08	< LOD	n.d.	n.d.
Mg	0.135 ± 0.03	< LOD	n.d.	n.d.
Al	0.27 ± 0.08	< LOD	n.d.	n.d.
S	10.7 ± 1.8	11.0 ± 1.1	10	103
K	1.04 ± 0.20	< LOD	n.d.	n.d.
Ca	5.1 ± 1.1	5.43 ± 0.32	6	107
Ti	8.6 ± 1.6	8.46 ± 1.69	20	98
V	1.30 ± 0.17	1.22 ± 0.13	11	94
Cr	1.39 ± 0.20	1.56 ± 0.07	4	112
Mn	0.094 ± 0.015	< LOD	n.d.	n.d.
Fe	28 ± 4	30.2 ± 2.7	9	108
Co	0.143 ± 0.017	0.146 ± 0.018	12	102
Ni	5.6 ± 0.6	5.8 ± 0.34	6	103
Cu	0.067 ± 0.012	0.063 ± 0.008	14	94
Zn	0.070 ± 0.020	< LOD	n.d.	n.d.
As (i)	0.016 ± 0.007	0.010 ± 0.001	6	65
Cd (i)	0.0022 ± 0.0019	< LOD	n.d.	n.d.
Pb	0.052 ± 0.028	< LOD	n.d.	n.d.

(r): Reference value. (i): Information value. n.d.: not determined. The measured values were obtained from three repeated analyses

To verify the applicability of the metal components in rCF derived from multi-material-structured CFRPs with metal materials and metal catalysts used for the extraction of CF during chemical recycling operations [16], we analyzed steel CRMs and metal oxides as model refractory metal components of rCF. The maximum amount of the inorganic components remaining in rCF samples was assumed to be 50% (w/w) of the rCF sample, corresponding to a sample-to-reagent ratio of 0.001 (w/v), i.e., 6 mg/6 ml. For a stainless steel CRM (JSS 651 − 16), the six target elements (Al, Cr, Fe, Co, Ni, and Cu) and additional 2 elements (V and Mn) were successfully determined (Table 5). For a carbon steel CRM (JSS 171-8), six target elements (Al, Ca, Ti, Cr, Fe, Ni) and additional 2 elements (Mn and As) were successfully determined. The RSD of Ca was slightly high (18%) because of the low concentration after the high-ratio dilution of the digested solution because of the extremely high concentration of iron (99%) for ICP-MS analysis. It is also noted that in the ICP-MS measurement of Al in the digested solution, the interference from the divalent ions of ^54^Fe, which is present at extremely high concentrations, caused significantly elevated values when using single MS detection. MS/MS measurements were used to separate the divalent ions of ^54^Fe, thereby improving the measurement values (Table 5). On the other hand, conventional closed-vessel microwave-assisted digestion methods at low-temperatures (160–200 °C) used a mixture containing phosphoric acid, sulfuric acid, or perchloric acid in addition to HF for complete dissolution and determination of 8 elements (S, V, Cr, Mn, Co, Ni, Mo and W) [10], 11 elements (B, Al, Si, P, Ti, V, Mn, Co, Cu, Mo and W) [11], and 9 elements (Al, Ca, Mg, Mn, Cr, Ni, Cu, Zn and Co) [12]. A pressurized-cavity microwave-assisted acid digestion using a mixture containing HF, nitric acid and hydrochloric acid with elevating temperature at 240 °C determined 7 elements (Cr, Mo, Ni, V, Mn, Cu and Co) [13]. When the 13 elements in this study were targeted, the proposed HF-free method was less hazardous and simpler than conventional methods. We also analyzed the metal oxides of the seven target elements (Al, Ti, Cr, Fe, Ni, Cu, and Zn). The determinations of six metals (Al, Cr, Fe, Ni, Cu, and Zn) were enabled even under conditions with half the high concentration of rCF samples (Table 5). In particular, decomposition of the highly refractory oxides of Cr and Fe required an elevated temperature condition at 280 °C, whereas the decomposition was incomplete under a lower temperature (220 °C) condition: the measured-calculated concentration ratios decreased to 57% and 44% (*n* = 1), respectively. On the other hand, aluminum oxide was decomposed and the aluminum was successfully determined even at the lower temperature condition (the concentration ratio of Al was 101% (*n* = 1)). The decomposition of titania was limited even at the high temperature condition at 280 °C. Based on the dissolution concentration value of the more refractory rutile-type titania, the upper limit of dissolution and determination of Ti in rCF by the proposed HF-free digestion method with an rCF sample-to-reagent ratio of 0.002 (w/v) was estimated to be 890 mg/kg. (1,780 mg/kg× 50%(w/w) = 890 mg/kg) The results for titanium in rCF1 and rCF2 samples obtained using the proposed method (429 and 25 mg/kg, respectively; see Table 1) are considered to be reasonable because the resultant concentrations were below the upper limit of dissolution for highly refractory rutile-type and there was also good agreement with using the HF-added digestion method (Table 1). Similarly, the Ti measurement results for the aforementioned CRMs (JSS 171-8 steel, BAM-0009 graphite, and NIST 1632d coal as shown in Tables 4 and 5) showed good agreement with the certified values, which were below the upper limit for the rutile type.

**Table 5:** Analyses of (a) steel certified reference materials (JSS 651 − 16 and 171-8) and (b) metal oxides as metallic analogues of rCF components

(a)				
Element	Certified value ± uncertainty (mg/kg)	Measured average value ± SD (mg/kg)	RSD (%)	Measured average value/certified value (%)
*JSS 651 − 16*				
Al	17.2 ± 3.2	15.5 ± 0.7^*a*^	5	90
		26.0 ± 10.9^*b*^	42	151
		143 ± 52^*c*^	36	830
V	746 ± 13	735 ± 5	1	99
Cr	181,200 ± 400	183,000 ± 3,000	2	101
Mn	9,250 ± 60	9,370 ± 120	1	101
Fe	712,890^*d*^	729,000 ± 7000	1	102
Co	2,170 ± 30	2,130 ± 30	2	98
Ni	80,400 ± 300	80,300 ± 1100	1	100
Cu	3,502 ± 54	3,450 ± 30	1	99
*JSS 171-8*				
Al	374 ± 6	375 ± 25^*b*^	7	100
		1,130 ± 1,150^*c*^	102	301
Ca	28 ± 2	27.4 ± 5.0	18	98
Ti	300 ± 6	291 ± 11	4	97
Cr	750 ± 20	733 ± 13	2	98
Mn	4,000 ± 30	3,970 ± 150	4	99
Fe	991,000^*d*^	1,030,000 ± 21,000	2	104
Ni	1,010 ± 10	937 ± 11	4	97
As	11 ± 2	10.3 ± 0.7	7	94

(b)					
Metal oxides	Target element	Calculated value (mg/kg)	Measured average value ± SD (mg/kg)	RSD (%)	Measured average value /calculated value (%)
α-alumina	Al	529,000	506,000 ± 44,000	9	96
titania (rutile-type)	Ti	599,000	1,790 ± 60	3	0.30
titania (anatase-type)	Ti		2,140 ± 80	4	0.36
chromium(III) oxide	Cr	684,000	653,000 ± 32,000	5	95
iron(III) oxide (hematite)	Fe	699,000	709,000 ± 51,000	7	101
nickel(II) oxide	Ni	786,000	743,000 ± 1,000	0.1	95
copper(II) oxide	Cu	799,000	807,000 ± 19,000	2	101
zinc oxide	Zn	803,000	816,000 ± 38,000	5	102

*a*: Determination was performed in H_2_ gas mode using MS/MS. *b*: Determination was performed using He gas mode with MS/MS. *c*: Determination was performed by He gas mode with single MS. *d*: Calculated values of iron were defined by subtracting the total concentration of impurity elements certified in the CRMs. The sample amount used was equal to 50% (w/w) of rCF sample amount. The measured values were obtained from three repeated analysis.

## Conclusion

The proposed HF-free pressurized-cavity microwave-assisted acid digestion combined with ICP-MS enabled the determination of 13 impurity elements (Na, Mg, Al, K, Ca, Ti, Cr, Fe, Co, Ni, Cu, Zn, and Pb) in the rCF. Increasing the temperature up to 280 °C especially promoted the dissolution of Na, Mg, Al, Ca, and Co, which indicates to be refractory chemical forms. The validity of the proposed method was verified through its agreement with the analytical results obtained using the HF‑added method, given the unavailability of appropriate CRMs. This agreement indicates that the HF‑free dissolution was sufficient for the multi‑element determination, including for the elements bound to silica. Furthermore, the proposed method was validated by analyzing carbon‑ and metal‑based CRMs with refractory characteristics, which were used as analogs of rCF components. However, the upper limit of titanium quantification is low, and if it is the highly refractory rutile chemical form, it is considered to be 890 mg/kg. In terms of operation, the proposed method requires only a one-step digestion without HF, which is simpler, less hazardous, and lowered LOQs for 10 of the 13 elements compared to the HF-added digestion method. The production and utility of rCF in CF recycling systems are expanding, and we believe that the established method will be useful for evaluating the sustainability and environmental responsibility of rCFs and will also make the analytical method less hazardous.

## Supplementary Information

Below is the link to the electronic supplementary material.

Supplementary Material 1

## Data Availability

Data are available on reasonable requests.
