# Supplementary material for: A simple hydrofluoric acid-free pressurized-cavity microwave-assisted acid digestion method for determination of impurity elements in recycled carbon fibers with ICP-MS
Source: Anal Sci. 2026 Mar 5;42(4):257–67. doi: 10.1007/s44211-026-00886-1 (PMC13013236; doi:10.1007/s44211-026-00886-1)
Supplement: Supplementary file 1 — Supplementary Material 1 [file 44211_2026_886_MOESM1_ESM.pdf]

Supplementary Information

Title

A simple hydrofluoric acid-free pressurized-cavity microwave-assisted acid digestion method for determination of impurity elements in recycled carbon fibers with ICP-MS

Tetsuya Nakazato* and Yoshiki Makino

*: corresponding author

Environmental Management Research Institute, National Institute of Advanced Industrial Science and Technology (AIST), 16-1 Onogawa, Tsukuba, Ibaraki 305-8569, Japan
Email: tet.nakazato@aist.go.jp

Table S1 Operating conditions of ICP-MS

Instrument	Agilent 8900
RF power	1550 W
Plasma gas flow rate	Ar 15 L min^{-1}
Auxiliary gas flow rate	Ar 0.9 L min^{-1}
Carrier gas flow rate	Ar 1 L min^{-1}
Make-up gas flow rate	Ar 0.1 L min^{-1}
Collision/reaction gas flow rates	
Helium-mode	He 5 ml min^{-1}
Hydrogen-mode	H_2 7 ml min^{-1}
Oxygen-mode	O_2 0.45 ml min^{-1}
Integration time	0.1 s
Measured isotopes	
at helium-mode with single MS	^{23}Na, ^{24}Mg, ^{27}Al, ^{49}Ti, ^{51}V, ^{52}Cr, ^{55}Mn, ^{56}Fe, ^{59}Co, ^{60}Ni ^{63}Cu, ^{66}Zn, ^{111}Cd, ^{208}Pb
at helium-mode with MS/MS	^{27}Al (mass filters Q1 and Q2: m/z 27)
at hydrogen-mode with MS/MS	^{27}Al (Q1 and Q2: m/z 27), ^{39}K (Q1and Q2: m/z 39), ^{40}Ca (Q1 and Q2: m/z 40), ^{80}Se (Q1 and Q2: m/z 80)
at oxygen-mode with MS/MS	S as ^{32}S^{16}O (mass filter Q1: m/z 32; Q2: m/z 48), As as ^{75}As^{16}O (mass filter Q1: m/z 75; Q2: m/z 91)
Internal standard	^{115}In

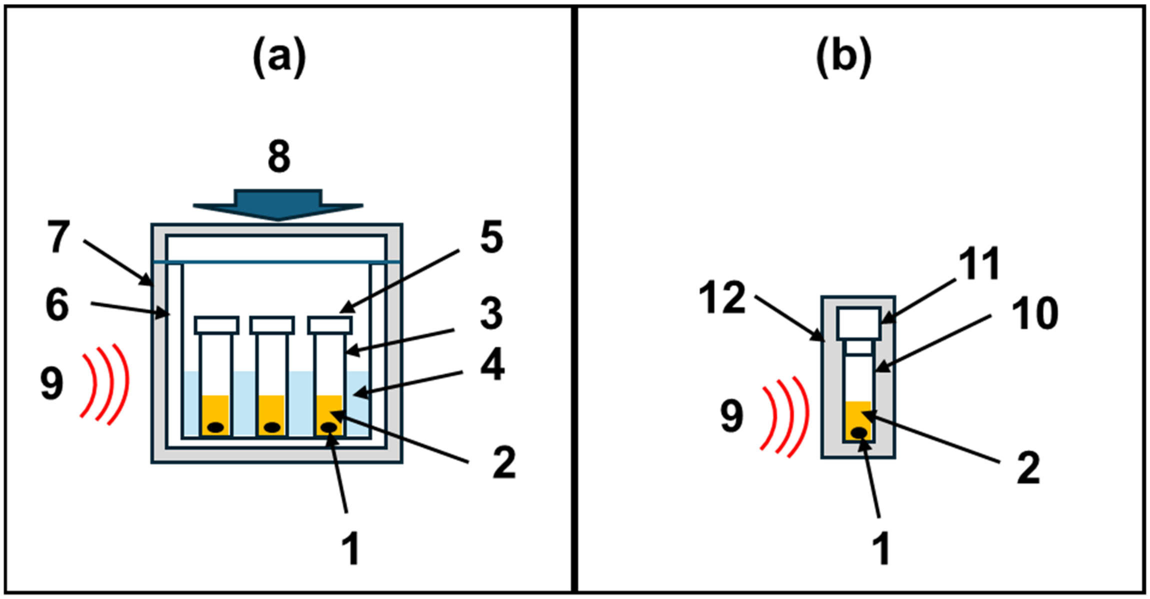

Figure S1 Conceptual diagrams of (a) pressurized-cavity and (b) closed-vessel microwave digestion system

The components are labeled as follows: 1, sample; 2, acids; 3, digestion vessel; 4, load solution; 5, cap; 6, liner; 7, pressurized cavity; 8, pressing unit using N_2 gas; 9, microwave; 10, closed-vessel; 11, seal cap; 12, protection jacket.

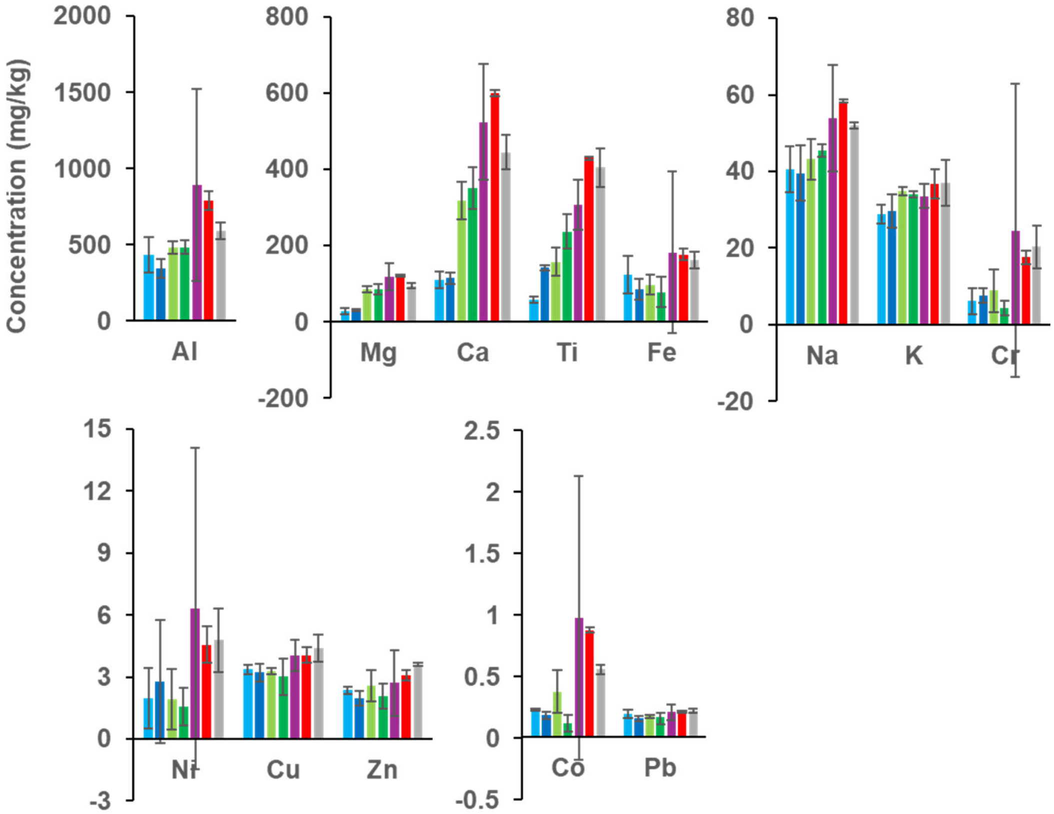

Figiure S2 Dissolution of impurity elements from rCF1 by HF-free microwave-assisted acid digestion using pressurized-cavity and closed-vessel systems

The unpulverized sample was digested with the closed-vessel system (210–230 °C) at sample-to-reagent ratios of 0.017 (w/v) (light blue) and 0.008 (blue), with the pressurized-cavity system (250 °C) at 0.008 (light green) and 0.002 (green), and with the pressurized-cavity system (280 °C) at 0.002 (purple). The pulverized sample was digested with the pressurized-cavity system at 280 °C (red) and with the closed-vessel system at 210–230 °C (gray) at 0.002.
